# Combined Stimulation of IL-2 and 4-1BB Receptors Augments the Antitumor Activity of E7 DNA Vaccines by Increasing Ag-Specific CTL Responses

**DOI:** 10.1371/journal.pone.0083765

**Published:** 2013-12-31

**Authors:** Ha Kim, Byungsuk Kwon, Jeong-Im Sin

**Affiliations:** 1 Department of Microbiology, School of Medicine, Kangwon National University, Chuncheon, Gangwon-do, Korea; 2 School of Biological Sciences, University of Ulsan, Ulsan, Korea; Mie University Graduate School of Medicine, Japan

## Abstract

Human papillomavirus (HPV) infection is a major cause of cervical cancer. Here, we investigate whether concurrent therapy using HPV E7 DNA vaccines (pE7) plus IL-2 vs. IL-15 cDNA and anti-4-1BB Abs might augment antitumor activity against established tumors. IL-2 cDNA was slightly better than IL-15 cDNA as a pE7 adjuvant. Co-delivery of pE7+IL-2 cDNA increased tumor cure rates from 7% to 27%, whereas co-delivery of pE7+IL-2 cDNA with anti-4-1BB Abs increased tumor cure rates from 27% to 67% and elicited long-term memory responses. This increased activity was concomitant with increased induction of Ag-specific CTL activity and IFN-γ responses, but not with Ag-specific IgG production. Moreover, the combined stimulation of IL-2 and 4-1BB receptors with rIL-2 and anti-4-1BB Abs resulted in enhanced production of IFN-γ from Ag-specific CD8+ T cells. However, this effect was abolished by treatment with anti-IL-2 Abs and 4-1BB-Fc, suggesting that the observed effect was IL-2- and anti-4-1BB Ab-specific. A similar result was also obtained for Ag-specific CTL activity. Thus, these studies demonstrate that combined stimulation through the IL-2 and 4-1BB receptors augments the Ag-specific CD8+ CTL responses induced by pE7, increasing tumor cure rates and long-term antitumor immune memory. These findings may have implications for the design of DNA-based therapeutic vaccines against cancer.

## Introduction

Human papillomavirus (HPV) infection is a primary cause of cervical cancer. Presently, Gardasil™ (Merck) and Cervarix™ (GSK) have been licensed as prophylactic vaccines against HPV infection. These vaccines are estimated to reduce the incidence of cervical cancer, but they are not effective in treating existing cervical cancer or its precancerous diseases (reviewed in [1], [2]). HPV E6- or E7-specific CTL responses have been reported to be critical in eliminating HPV-associated cervical intraepithelial neoplasia and cervical cancer in both animal and human studies (reviewed in [1], [2]). To date, numerous E7 DNA vaccine approaches (such as codon optimization, antigen targeting modification, co-injection of adjuvants, etc.) have been shown to augment Ag-specific CTL responses [3]–[5]. Similar to reports in other animal models [6], [7], we also found that delivery of E7 DNA vaccines using *in vivo* electroporation (EP) is more effective at inducing therapeutic antitumor activity through increased antigen production at and attraction of immune cells to the DNA injection sites [8]. A recent phase I clinical study showed that HPV 16 and 18 E6 and E7 DNA vaccines delivered by EP induced a significant level of Ag-specific humoral and cellular responses including CTL responses [9]. Collectively, these studies show that using EP as a DNA delivery method has a high potential to augment Ag-specific immune responses in humans and small animals.

IL-2 and IL-15 are known to share receptor subunits and have functional similarity in T cells [10], [11]. These cytokines activate the T cell processes of proliferation, cytokine production and survival through the activation of signal transducers and activators of transcription (STAT) 3 and STAT5 proteins [12]. Consistent with these known functions, plasmid DNAs expressing IL-2 and IL-15 enhance vaccine-induced T cell responses [13], [14]. Moreover, IL-2 has been approved for clinical use in patients with metastatic renal cell carcinoma and melanoma [15], [16] and has been tested with adoptively transferred immune cells for treating patients with melanoma [17]. In contrast, 4-1BB (CD137) is a member of the tumor necrosis factor receptor superfamily expressed on the surface of activated T cells, but not resting T cells [18], [19]. 4-1BB activation provides a potent costimulatory signal to CD8+ and, to a lesser extent, CD4+ T cells [20]. Agonistic anti-4-1BB Abs have been reported to enhance tumor rejection and increase tumor-specific cytotoxicity in numerous studies [21]–[23]. In our recent study, large established subcutaneous B16 melanomas were effectively controlled by combined therapy using Trp2 peptide vaccines with a Toll-like receptor 9 agonist (CpG-ODN) and anti-4-1BB Abs [24]. In this context, it can be speculated that combined stimulation through IL-2, IL-15 and 4-1BB receptors may enhance Ag-specific CD8+ CTL responses induced by E7 DNA vaccines, thereby conferring more effective tumor control in an HPV E7-expressing tumor model.

## Materials and Methods

### Animals

Six week-old female C57BL/6 mice were purchased from Daehan Biolink (Chungbuk, Korea). The mice were cared for under the guidelines of the Kangwon Institutional Animal Care and Use Committee-approved protocols (KW-130419-1). This was approved by the Animal Care and Use Committee of Kangwon National University.

### Reagents and Treatment of Mice

For intramuscular (IM)-electroporation (EP) delivery, mice were injected intramuscularly with 50 µg of E7 DNA vaccines (pcDNA3-Sig/sE7/LAMP, pE7) [5] per mouse with or without 10 µg of IL-2 and IL-15 cDNAs [14] in a final volume of 50 µl of phosphate-buffered saline (PBS) using a 31-gauge needle (BD, Franklin Lakes, NJ). Specifically, we tested plasmid DNAs encoding a full length of IL-2 and IL-15 in this study. The injections were followed by EP at 0.2 volts for 4 sec using Cellectra® of VGX International Inc./Inovio in accordance with the manufacturer’s protocol. Plasmid DNA was produced in bacteria and purified by endotoxin-free Qiagen kits according to the manufacturer’s protocol (Qiagen, Valencia, CA). For adoptive transfer of anti-4-1BB Abs, the animals were injected intraperitoneally (i.p.) with 100 µg of anti-4-1BB Abs unless mentioned otherwise. Anti-4-1BB Abs were generated from hybridoma cells (3H3), which were a kind gift of R. Mittler (Emory University, Atlanta, GA). Specifically, pristane-primed nude mice were injected with 3H3 cells for ascites fluid production from which anti-4-1BB Abs were purified using a protein G column (Sigma-Aldrich, St. Louis, MO). Control rat immunoglobulin G (IgG) was purchased from Sigma-Aldrich.

### *In vivo* CTL Lytic Activity Assay

Spleen cells from naïve mice were treated with red blood cell lysis buffer (Sigma). One fraction of the splenocytes was then pulsed with 5 µg of E7 peptides {containing the major histocompatibility complex (MHC) Class I epitope at amino acids 49 to 57} in cRPMI for 60 min at 37°C, while the other fraction was left un-pulsed. To generate peptide-pulsed cells with high carboxyfluorescein diacetate succinimidyl ester (CFSE), the peptide-pulsed splenocytes were incubated with 20 µM CFSE in RPMI (2.5% FBS) for 15 min. The un-pulsed cells were instead incubated with 2.5 µM CFSE in RPMI (2.5% FBS) for 15 min to generate non-peptide-pulsed cells with low CFSE. The cells were then washed 3 times with PBS to remove unbound CFSE. Finally, an equal number of pulsed and un-pulsed cells (a total of 2×10^7^ cells/0.4 ml/mouse) were injected intravenously (i.v.) into the tested mice. After 8–10 h, the mice were sacrificed and the spleens were collected. After lysing the red blood cells, the splenocytes were analyzed directly for the two cell populations with CFSE staining (CFSE low versus CFSE high) using a flow cytometer (BD). The percentage of lysed cells (%lysis) was calculated as 100×{1−(*r*_unprimed_/*r*_primed_)}. The ratio (*r*) was calculated as %CFSE^low^/%CFSE^high.^

### IFN-γ Assay

A 1 ml aliquot containing 6×10^6^ splenocytes was added to each well of 24-well plates containing 1 µg of E7 peptides (containing the MHC Class I epitope at amino acids 49 to 57) or Trp2 peptides as a control. In one experiment, increasing doses of recombinant IL-2 (BD) and anti-4-1BB Abs were also added to the splenocytes to analyze IFN-γ levels. For blocking experiments, recombinant IL-2 proteins and anti-4-1BB Abs were incubated for 1 h 30 m with 5 µg anti-IL-2 Abs per ml and 5 µg 4-1BB-Fc proteins [25] per ml and then added to the splenocytes. The E7 CTL peptides (RAHYNIVTF) and Trp2 peptides (SVYDFFVWL) were purchased from Peptron, Taejon, Korea. After 3 days of incubation at 37°C in 5% CO_2_, cell supernatants were isolated and used to analyze IFN-γ levels, which was performed with commercial cytokine kits (BD) and by adding the extracellular fluids to IFN-γ-specific enzyme-linked immunosorbent assay (ELISA) plates.

### ELISA

ELISA to detect Abs against E7 protein was performed as previously described [26], [27]. In particular, for the determination of relative levels of E7-specific immunoglobulin G (IgG) subclasses, anti-murine IgG1, IgG2a, IgG2b and IgG3 conjugated with horseradish peroxidase (HRP) (Zymed, San Francisco, Calif.) were substituted for anti-murine IgG-HRP. For this assay, recombinant E7 protein [26] (1 µg/ml in PBS) was used as a coating antigen. For detection of IL-2 and IL-15, 100 µl of sera, cell supernatants and cell lysates were added to IL-2- and IL-15-specific ELISA plates (Biolegend, San Diego, CA) in accordance with the manufacturer’s protocols. Specifically, rhabdomyosarcoma (RD) cells (4×10^6^ cells/plate) were transfected with 4 µg of IL-2 and IL-15 plasmid DNAs using JetPEI™ transfection reagents (Polyplus-Transfection Inc., New York). Two days following DNA transfection, cell supernatants were collected and used to analyze cytokine levels. The remaining cells were resuspended in 500 µl of PBS and lysed by 3 freeze-thaw cycles. The cell lysates were subsequently used to analyze cytokine levels.

### Flow Cytometry

To analyze CD44^high^CD8 positive T cell populations, tumor-cured mice were sacrificed and the spleens were removed for immune cell isolation. The isolated cells were incubated with APC-labeled anti-CD44 (BD) and PE-labeled anti-CD8 Abs (BD) for fluorescence-activated cell sorting (FACS) analysis.

### Tumor Cell Challenge and Antitumor Therapeutic Studies

For the antitumor therapeutic studies, 2×10^5^ TC-1 cells per mouse were injected subcutaneously (s.c.) into the right flank of C57BL/6 mice. When the tumor size reached approximately 5 mm, the animals were injected with E7 DNA vaccines and/or plasmid DNAs expressing IL-2 and IL-15 by IM-EP. The animals were also injected i.p. with anti-4-1BB Abs. For tumor re-challenge studies, 2–4×10^5^ TC-1 cells per mouse were injected s.c. into the left flank of C57BL/6 mice. TC-1 tumor cells were previously tested [3], [4] and kindly provided by T.-C. Wu (Johns Hopkins University, Baltimore, MD). The TC-1 cells were grown in cRPMI supplemented with 400 µg per ml of G418, washed 2 times with PBS and injected into mice. The mice were monitored twice per week for tumor growth. The tumor growth was measured in mm using a caliper, and was recorded as mean diameter {longest surface length (a) and width (b), (a+b)/2}. Tumor-cured animals were denoted as those showing complete tumor regression for 120 days after treatment. The mice were euthanized when the mean diameter of the tumor exceeded 20 mm.

### Statistical Analysis

Statistical analysis was performed by independent *t* test, one-way ANOVA and Chi square test (Fisher’s exact test) using the SPSS 17.0 software program. The values of the experimental groups were compared with the values of the control group. Any *p* values <0.05 were considered to be significant.

## Results

### An E7 DNA Vaccine Combined with IL-2 or, to a Lesser Degree, IL-15 cDNA Induced Greater Antitumor Therapeutic Activity than an E7 DNA Vaccine Alone

**Figure 1:**
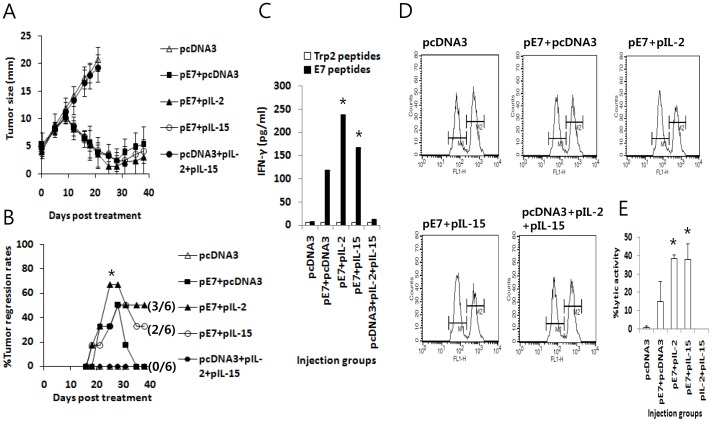
Antitumor therapeutic activity (A,B), Ag-specific IFN-γ production *in vitro* (C) and Ag-specific CTL lytic activity *in vivo* (D,E) in animals injected with E7 DNA vaccines in combination with IL-2 and IL-15 cDNAs. Each group of mice (n = 6) was challenged s.c. with 2×10^5^ TC-1 cells per mouse. When the tumor size reached approximately 5 mm, animals were injected by IM-EP with 50 µg pE7 in the presence or absence of 10 µg of IL-2 and IL-15 cDNA per mouse in a final volume of 50 µl at 0, 4, and 11 days. The tumor sizes were measured over time (A). The values and bars represent the mean tumor size and SD, respectively. Simultaneously, animals showing complete tumor regression were counted over the time points and the % tumor regression rates were calculated (B). The numbers in (/) denote the number of mice showing complete tumor regression for 120 days after treatment/the number of mice tested. *p<0.05 using Chi-square test compared to pE7+pcDNA3. (C) Each group of mice (n = 5) was injected by IM-EP with 50 µg of E7 DNA vaccine (pE7) along with 10 µg of IL-2 and IL-15 cDNA per mouse in a final volume of 50 µl at 0 and 1 weeks. The animals were sacrificed at 2 weeks and isolated splenocytes (6×10^6^ cells/ml) were stimulated *in vitro* for 3 days with 1 µg/ml E7 CTL peptides or control Trp2 peptides. The cell supernatants were used for measuring IFN-γ. The values and bars represent the mean IFN-γ level and SD, respectively. (D) Similar experiment as in panel (C), except that animals were tested for *in vivo* CTL lytic activity at 2 weeks. For this test, E7 peptide-pulsed (CFSE high) and un-pulsed (CFSE low) splenocytes were injected i.v. into the immunized mice, as described in “Materials and Methods”. The mice were sacrificed after 8 h and splenocytes were analyzed by FACS to measure the level of CFSE-labeled cells in each subset. M1, un-pulsed CFSE low population; M2, E7-pulsed CFSE high population. (E) shows the mean % CTL lytic activity of each test group and SD. This study was repeated twice with similar results. *p<0.05 using one-way ANOVA compared to pE7+pcDNA3.

To test whether the combined administration of E7 DNA vaccines (pE7) with either IL-2 or IL-15 cDNA induces greater antitumor therapeutic activity than pE7 alone, we injected tumor (5 mm)-bearing animals with 50 µg of pE7 with either 10 µg of IL-2 (pIL-2) or IL-15 (pIL-15) cDNA at 0, 4 and 11 days and checked tumor growth patterns over the time points following treatment. As shown in Fig. 1A, animals treated with pE7+pIL-2 or, to a lesser degree, with pE7+pIL-15 showed enhanced tumor growth suppression over time when compared to those treated with pE7 alone. Animals treated with pE7 alone showed improved tumor growth suppression when compared to control mice. Fig. 1B shows the percentage of animals showing complete tumor regression over the time points measured following treatment. Specifically, 3 of 6 animals (50%) treated with pE7+pIL-2 showed complete tumor cure, whereas 2 of 6 animals (33%) treated with pE7+pIL-15 showed complete tumor cure. In contrast, none of the animals (0%) treated with pE7 alone showed complete tumor cure. At this point, we injected the DNA at 0, 4 and 11 days, as this injection time frame was previously found to induce better tumor control in the established TC-1 model [8]. We also chose 10 µg of IL-2 or IL-15 cDNA for injection with E7 DNA vaccines because 20 µg of IL-2 or IL-15 cDNA injected by IM-EP made the treated animals sick and moribund following treatment. These data collectively suggest that the combination of an E7 DNA vaccine with IL-2 or, to a lesser degree, IL-15 cDNA may induce increased antitumor activity against established TC-1 tumors than an E7 DNA vaccine alone.

### An E7 DNA Vaccine Combined with IL-2 or, to a Lesser Degree, IL-15 cDNA Induced Increased Ag-specific IFN-γ Production *in vitro* and CTL lytic Activity *in vivo* Compared to an E7 DNA Vaccine Alone

**Figure 2:**
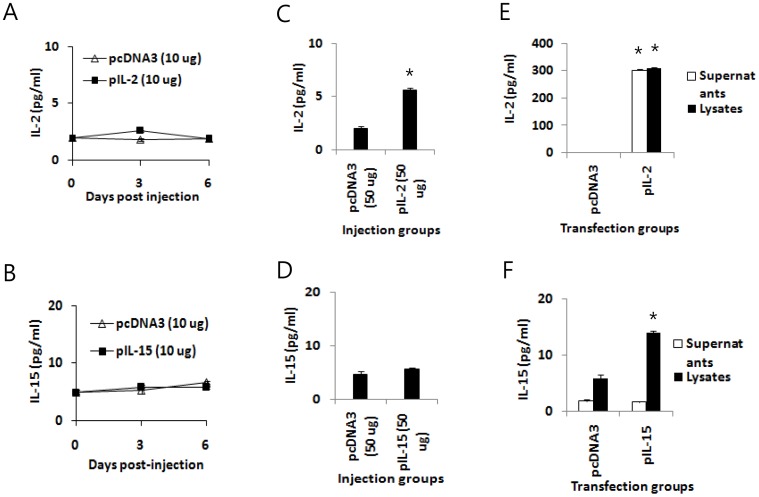
Expression of IL-2 and IL-15 in animals and cell cultures by plasmid DNAs. (A,B) Each group of mice (n = 5) was injected by IM-EP with 10 µg of IL-2 cDNA (pIL-2) (A) and IL-15 cDNA (pIL-15) (B) per mouse in a final volume of 50 µl. The animals were bled on days 3 and 6 post-injection. (C,D) Each group of mice (n = 5) was injected by IM-EP with 50 µg of pIL-2 (C) and pIL-15 (D) per mouse in a final volume of 50 µl. The animals were bled on day 3 post-injection. The sera were tested for levels of IL-2 and IL-15. (E,F) RD cells (4×10^6^ cells/plate) were transfected with 4 µg of pIL-2 (E) and pIL-15 (F). Two days following transfection, cell supernatants and lysates were collected for measuring the levels of IL-2 and IL-15. *p<0.05 using independent *t* test compared to pcDNA3.

Naïve mice were immunized with pE7 plus pIL-2 or pIL-15 to measure Ag-specific IFN-γ induction levels from the immune cells of the immunized mice. The animals were sacrificed after immunization, and the spleens were removed for immune cell isolation. The isolated cells were stimulated *in vitro* with E7 CD8+ CTL peptides. As shown in Fig. 1C, when immune cells from animals immunized with pE7+pIL-2 were stimulated *in vitro* with E7 peptides, they produced the highest levels of IFN-γ. The next highest were produced by immune cells from animals immunized with pE7+pIL-15, followed by cells from animals immunized with pE7 alone. IFN-γ production from Ag-specific CD8+ T cells by pE7 immunization was increased over two-fold by the presence of IL-2 cDNA. We also measured Ag-specific CTL lytic activity in animals immunized with E7 DNA vaccines (pE7) in the presence of either IL-2 cDNA (pIL-2) or IL-15 cDNA (pIL-15). As shown in Fig. 1D and E, animals immunized with pE7+pIL-2 or pE7+pIL-15 had greater Ag-specific CTL lytic activity compared to those immunized with pE7 alone. These *in vitro* IFN-γ production and *in vivo* CTL lytic activity profiles are consistent with the therapeutic antitumor activity we observed in Fig. 1A and B. At the tested cytokine DNA dose (10 µg per mouse), however, we were unable to detect IL-2 and IL-15 in sera (Fig. 2A,B). IL-2 was detectable in sera after injection of 50 µg IL-2 plasmid DNAs (Fig. 2C), whereas IL-15 was undetectable in sera even after injection of 50 µg IL-15 plasmid DNAs (Fig. 2D). In the subsequent DNA transfection study, IL-2 was detectable in both cell supernatants and cell lysates (Fig. 2E). On the other hand, IL-15 was detectable only in cell lysates but not in cell supernatants (Fig. 2F), suggesting that IL-15 might be unstable and/or cell surface-attached.

### Adoptive Transfer of Anti-4-1BB Abs Dramatically Augmented the Antitumor Efficacy of the E7 DNA Vaccine Regimen (E7 DNA Vaccines+IL-2 cDNA) and Induced Potent Long-term Antitumor Memory Responses

**Figure 3:**
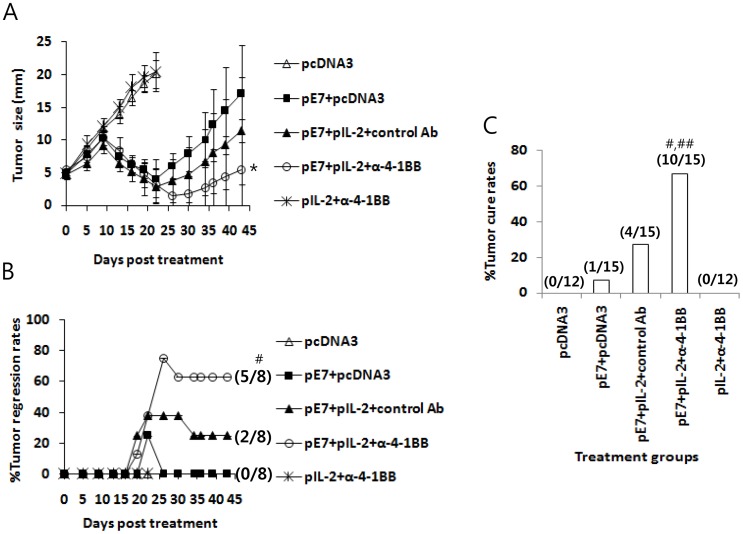
Antitumor therapeutic activity of the E7 DNA vaccine regimen (E7 DNA vaccines+IL-2 cDNA) plus anti-4-1BB Abs. (A) Each group of mice (n = 6–8) was challenged s.c. with 2×10^5^ TC-1 cells per mouse. When the tumor sizes reached approximately 5 mm, the animals were injected by IM-EP with 50 µg of E7 DNA vaccines (pE7) plus 10 µg of IL-2 cDNA (pIL-2) per mouse in a final volume of 50 µl at 0, 4, and 11 days. The animals were also injected i.p. with 100 µg of anti-4-1BB and control rat Abs at 0, 1 and 2 weeks. The tumor sizes were measured over the entire time course (A). The values and bars represent the mean tumor sizes and SD, respectively. Simultaneously, animals showing complete tumor regression were counted over the time points and the % tumor regression rates were calculated (B). The numbers in (/) denote the number of mice showing complete tumor regression for 120 days after treatment/the number of mice tested. (C) The percentage of mice showing complete tumor regression for 120 days after treatment. The numbers in (/) denote the number of mice showing complete tumor regression for 120 days after treatment/the total number of mice tested in two separate studies. *p<0.05 using one-way ANOVA compared to pE7+pcDNA3. #p<0.05 using Chi-square test compared to pE7+pcDNA3. ##p<0.05 using Chi-square test compared to pE7+pIL-2+control Abs.

**Figure 4:**
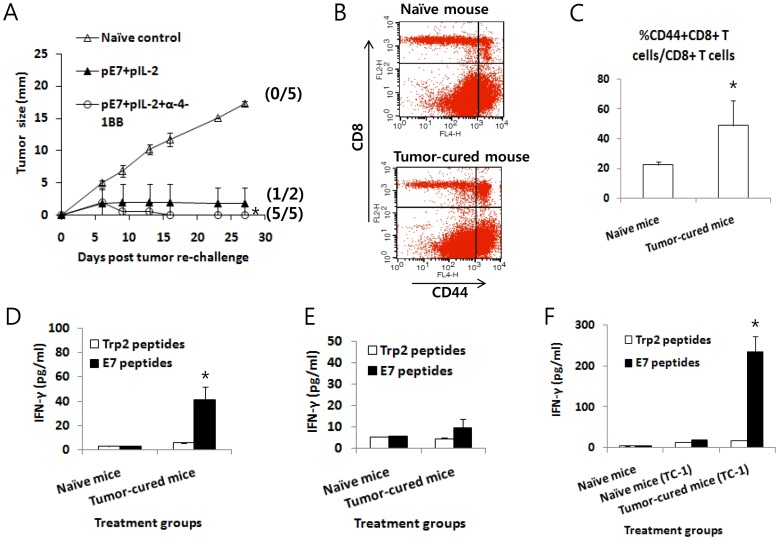
Induction of long-term antitumor memory responses by the E7 DNA vaccine regimen (E7 DNA vaccines+IL-2 cDNA) plus anti-4-1BB Abs. (A) Five tumor-cured mice treated with pE7+pIL-2+anti-4-1BB Abs (from Fig. 3B) and 2 tumor-cured mice treated with pE7+pIL-2+control Abs (from Fig. 3B) were re-challenged s.c. with 4 x 10^5^ TC-1 cells per mouse at 120 days post-first treatment. The tumor size was measured over time, and the mean tumor size was recorded. The values and bars represent the mean tumor sizes and SD, respectively. The numbers in (/) indicate the number of mice showing tumor regression for 270 days post-re-challenge/the number of mice tested. (B,C,D) Three of the 5 mice that received tumor cell re-challenge and rejected their tumors (from Fig. 4A) were sacrificed at 270 days post-tumor re-challenge and the spleens were removed for immune cell isolation. The immune cells were tested for the level of the CD44^high^CD8+ T cell population among CD8+ T cells (B,C) and for the level of IFN-γ induction (D). Fig. 4B shows one representative figure displaying the level of CD44^high^CD8+ T cells. Fig. 4C shows the mean percentage of CD44^high^CD8+ T cells in each group and SD. Fig. 4D shows IFN-γ levels after *in vitro* stimulation with either E7 or control Trp2 peptides. (E,F) In another set of mice from a tumor treatment study, 3 of 5 tumor-cured mice following treatment with E7 DNA+IL-2+anti-4-1BB were sacrificed at 120 days post-treatment and the spleens were removed for immune cell isolation. The immune cells were used for IFN-γ assays (E). The remaining two mice from Fig. 4E were re-challenged with TC-1 cells (2×10^5^ cells/mouse) at 138 days post-treatment in parallel with naïve mice. The mice were sacrificed at 18 days post-re-challenge and the spleens were removed for an IFN-γ assay (F). (E,F) show the IFN-γ levels in each group. The values and bars represent the mean IFN-γ levels of each test group and SD. *p<0.05 using one-way ANOVA compared to naïve control.

Previously, we observed that IL-2 cDNA was slightly better than IL-15 cDNA as an E7 DNA vaccine adjuvant in inducing both therapeutic antitumor and Ag-specific cellular responses. Therefore, we chose IL-2 over IL-15 for further testing. It is known that IL-2 activates CD8+ T cells through its receptor-mediated Janus kinase (JAK)/STAT signaling pathway [28], [29]. Anti-4-1BB Abs activate the CD8+ T cell processes of proliferation, cytokine production and survival through 4-1BB/TRAF (tumor necrosis factor receptor-associated factor)-mediated JNK (c-Jun N-terminal kinase)/stress-activated protein kinase, p38 MAPK (mitogen-activated protein kinase) and nuclear factor (NF)-kB signaling pathways [30], [31], and the TCR signaling pathway [32]. Thus, we hypothesized that additional 4-1BB signaling in conjunction with IL-2 receptor signaling might augment Ag-specific CD8+ T cell responses induced by E7 DNA vaccines through these two separate signaling pathways, thereby increasing antitumor therapeutic activity against established tumors. To test this hypothesis, we adoptively transferred anti-4-1BB Abs into tumor-bearing animals following treatment with the E7 DNA vaccine regimen {E7 DNA vaccines (pE7)+IL-2 cDNA (pIL-2)}. As shown in Fig. 3A, animals treated with pE7+pIL-2+control Abs displayed greater antitumor therapeutic activity compared to those treated with pE7 alone. However, concurrent treatment of tumor-bearing mice with pE7+pIL-2+anti-4-1BB Abs resulted in significantly higher therapeutic antitumor activity compared to treatment with pE7+pcDNA3. Fig. 3B shows the percentage of animals showing complete tumor regression over the time points following treatment with pE7+pIL-2+anti-4-1BB Abs. Animals treated with pE7+pIL-2+control Abs showed a 25% tumor cure rate (2/8), while those treated with pE7+pIL-2+anti-4-1BB Abs exhibited a 63% tumor cure rate (5/8), a 2.5-fold increase in tumor cure rates with the addition of anti-4-1BB Abs to the E7 DNA vaccine regimen. However, no mice treated with pE7 alone were cured (a 0% tumor cure rate; 0/6). Overall, the tumor cure rates for treated-animals were 0% for pcDNA3 (0/12), 7% for pE7+pcDNA3 (1/15), 27% for pE7+pIL-2+control Abs (4/15), 67% for pE7+pIL-2+anti-4-1BB Abs (10/15) and 0% for pIL-2+anti-4-1BB Abs (0/12) (Fig. 3C). These data demonstrate that the combined stimulation of IL-2 and 4-1BB receptors with IL-2 from IL-2 cDNA and anti-4-1BB Abs, respectively, might act in concert to increase therapeutic antitumor activity induced by E7 DNA vaccines in this tumor model. We also evaluated whether tumor-cured mice following treatment with pE7+pIL-2+control Abs and pE7+pIL-2+anti-4-1BB Abs may have developed long-term antitumor memory responses to parental TC-1 tumor cells. Following treatment with pE7+pIL-2+anti-4-1BB, five tumor-cured mice from Fig. 3B were re-challenged with TC-1 cells at 120 days post-first treatment, and they exhibited some tumor formation at an early time point that regressed thereafter (Fig. 4A). In contrast, of the 2 tumor-cured mice treated with pE7+pIL-2+control Abs from Fig. 3B, 1 showed incomplete tumor protection from tumor cell re-challenges. These data suggest that the combined use of IL-2 cDNA as a molecular adjuvant and anti-4-1BB Abs may lead to augmentation of long-term protective immunity in the form of antitumor memory responses. We subsequently tested whether these tumor-cured mice have memory cell phenotypes and IFN-γ memory responses to E7 antigens. For this testing, 3 of the 5 tumor-cured mice that previously received pE7+pIL-2+anti-4-1BB from Fig. 4A were sacrificed 270 days post-tumor cell re-challenge, and the spleens were removed for immune cell isolation, along with spleens from naïve control mice. The immune cells were measured for the percentage of CD44^high^CD8+ T cells among the total CD8+ T cells. As shown in Fig. 4B and C, naïve mice showed 22% CD44^high^CD8+ T cells among the total CD8+ T cells, while the tumor-protected mice displayed 49% CD44^high^CD8+ T cells among the total CD8+ T cells. This represents a more than 2-fold increase in CD44^high^CD8+ T cell populations, representative of the memory cell phenotype in tumor-cured mice. Immune cells from tumor-cured mice also showed a more dramatic level of IFN-γ responses to E7 CTL peptides *in vitro* compared to those of naïve mice showing a background level of IFN-γ production (Fig. 4D). CD44^high^CD8+ T cell phenotypes and IFN-γ memory responses were similarly observed in the 2 remaining tumor-cured mice from Fig. 4A in a separate assay (data not shown). In another set of tumor treatment studies, 3 of the 5 tumor-cured mice treated with pE7+pIL-2+anti-4-1BB Abs were sacrificed 4 months post-first treatment and the spleens were removed for immune cell isolation. The immune cells were then assessed for IFN-γ memory responses to E7 antigens and the levels of memory cell phenotypes. As shown in Fig. 4E, an insignificant level of IFN-γ production was detected when immune cells from the tumor-cured mice were stimulated with E7 antigens compared to responses from naïve mice. In these animal groups, there was no significant difference in the percentage of CD44^high^CD8+ T cells among the total CD8+ T cells (data not included). Because we were unable to see any significant difference in IFN-γ memory responses to E7 antigens between these 2 groups, we re-challenged the 2 remaining tumor-cured mice with TC-1 cells (2×10^5^ cells/mouse) at 138 days post-treatment in parallel with naïve mice. At 18 days following tumor cell re-challenge, the mice were sacrificed to measure IFN-γ responses (Fig. 4F). At this point, the tumor-cured mice formed tumors at an early time points, which regressed thereafter, whereas naïve mice formed tumors (tumor size, ≅13 mm). As shown in Fig. 4F, immune cells from tumor-regressed mice following tumor re-challenge showed a more dramatic level of IFN-γ production after *in vitro* stimulation with E7 peptides compared to cells from naïve mice showing a background level of IFN-γ production. This result shows that combined therapy using E7 DNA vaccines+pIL-2 in combination with anti-4-1BB Abs might augment Ag-specific long-term memory immune responses as well as antitumor memory activity. Taken together, these data suggest that the combined use of IL-2 cDNA with anti-4-1BB Abs might lead to augmentation of therapeutic antitumor activity induced by E7 DNA vaccines and long-term antitumor memory immunity in this TC-1 model.

### Adoptive Transfer of Anti-4-1BB Abs Significantly Inhibited Production of Ag-specific IgG and IgG2b Isotype Induced by E7 DNA Vaccination

**Figure 5:**
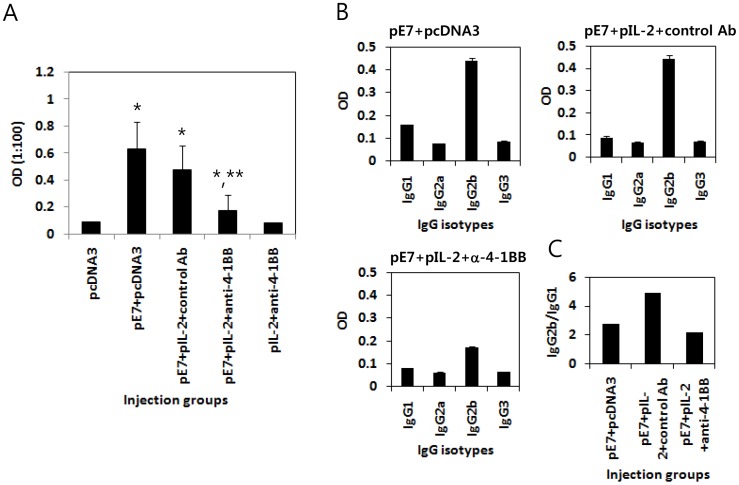
Inhibition of E7-specific IgG and IgG2b production by the E7 DNA vaccine regimen (E7 DNA vaccine+IL-2 cDNA) plus anti-4-1BB Abs. Each group of mice (n = 6–8) was challenged s.c. with 2×10^5^ TC-1 cells per mouse. When the tumor sizes reached approximately 5 mm, the mice were treated by IM-EP with 50 µg of E7 DNA vaccines plus 10 µg of IL-2 cDNA (pE7+pIL-2) at 0, 4 and 11 days. The animals were also injected i.p. with 100 µg of anti-4-1BB and control rat Abs at 0, 1 and 2 weeks. The mice were bled at 11 days following the last anti-4-1BB Ab injection, and sera in each group were equally pooled and diluted to 1∶100 for reaction with E7 proteins for ELISA. Samples were assayed in triplicate. The values of E7-specific IgG (A) and IgG subclasses (B), and bars represent the mean optical density and SD, respectively. (C) The IgG2b/IgG1 ratio was calculated by dividing the mean optical density of IgG2b by that of IgG1. *p<0.05 using one-way ANOVA compared to pcDNA3. **p<0.05 using one-way ANOVA compared to pE7+pcDNA3.

Subsequently, we measured the levels of Ag-specific antibodies in tumor-bearing animals following injection of E7 DNA vaccines (pE7)+IL-2 cDNA (pIL-2) in combination with anti-4-1BB Abs. As shown in Fig. 5A, tumor-bearing animals treated with pE7+pIL-2+anti-4-1BB Abs displayed significantly lower Ag-specific IgG responses compared to animals treated with pE7+pIL-2+control Abs or pE7 alone. However, there was no significant difference in Ag-specific IgG production between the animal groups treated with pE7 alone or pE7+pIL-2+control Abs. These data show that addition of anti-4-1BB Abs to the E7 DNA vaccine regimen may result in decreased production of Ag-specific IgG. Fig. 5B shows the production profiles of the Ag-specific IgG isotypes. Tumor-bearing animals treated with pE7+pIL-2+anti-4-1BB Abs also had significantly lower production of Ag-specific IgG2b isotypes compared to animals treated with either pE7+pIL-2+control Abs or pE7 alone. The ratios of IgG2b to IgG1 were 2.8 for pE7+pcDNA3, 4.9 for pE7+pIL-2+control Abs, and 2.2 for pE7+pIL-2+anti-4-1BB Abs (Fig. 5C).

### E7 DNA Vaccines+IL-2 cDNA+anti-4-1BB Abs Induced Significantly Higher Ag-specific CTL Lytic Activity *in vivo* Compared to Either E7 DNA Vaccines or E7 DNA Vaccines+IL-2 cDNA+control Abs

**Figure 6:**
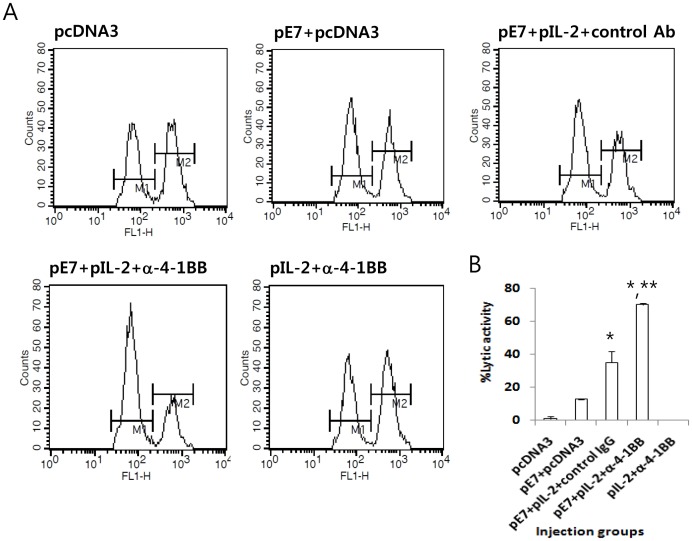
Evaluation of Ag-specific CTL lytic activity in animals injected with the E7 DNA vaccine regimen (E7 DNA vaccines+IL-2 cDNA) plus anti-4-1BB Abs. (A) Each group of mice (n = 5) was injected by IM-EP with 50 µg of E7 DNA vaccines (pE7) plus 10 µg of IL-2 cDNAs (pIL-2) per mouse in a final volume of 50 µl at 0 and 1 weeks. The animals were also injected i.p. with 100 µg of anti-4-1BB and control rat Abs at 0 and 1 weeks. The animals were tested for *in vivo* CTL lytic activity at 2 weeks. For this test, E7 peptide-pulsed (CFSE high) and un-pulsed (CFSE low) splenocytes were injected i.v. into the immunized mice, as described in “Materials and Methods.” The mice were sacrificed after 8 h and the splenocytes were analyzed by FACS to measure the level of CFSE-labeled cells in each subset. M1, un-pulsed CFSE low population; M2, E7-pulsed CFSE high population. (B) shows the mean % CTL lytic activity of each test group and SD. *p<0.05 using one-way ANOVA compared to pE7+pcDNA3. **p<0.05 using one-way ANOVA compared to pE7+pIL-2+control IgG.

Next, we measured Ag-specific CTL activity in mice injected with E7 DNA vaccines (pE7) plus IL-2 (pIL-2) and anti-4-1BB Abs. For this test, naïve animals were injected with pE7, pIL-2 and anti-4-1BB Abs, and then tested for CTL lytic activity. As shown in Fig. 6, animals injected with pE7+pIL-2+control Abs had significantly higher Ag-specific CTL lytic activity compared to animals injected with pE7 alone. However, animals injected with pE7+pIL-2+anti-4-1BB Abs showed significantly higher CTL lytic activity compared to animals injected with pE7+pIL-2+control Abs. Control mice and mice injected with pIL-2 plus anti-4-1BB Abs also exhibited a background level of CTL lytic activity *in vivo*. These data on *in vivo* CTL activity are consistent with the therapeutic antitumor activity observed in Fig. 3.

### Increased Production of IFN-γ from Ag-specific CD8+ T cells *in vitro* Required IL-2 and Anti-4-1BB Abs

**Figure 7:**
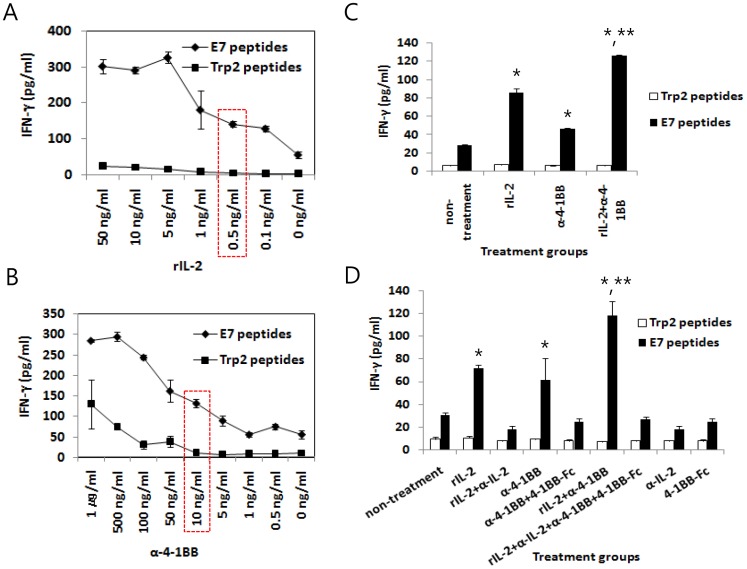
Increased production of IFN-γ from Ag-specific CD8+ T cells by treatment with IL-2 and anti-4-1BB Abs, and its blockade by treatment with anti-IL-2 Abs and 4-1BB-Fc. Mice were immunized by IM-EP with 50 µg of E7 DNA vaccines (pE7) at 0 and 1 weeks. At 2 weeks, the mice were sacrificed and then the spleens were removed for immune cell isolation. The immune cells (6×10^6^ cells/ml) were stimulated *in vitro* with either 1 µg/ml E7 CTL epitopes or control Trp2 peptides in the presence of an increasing dose of rIL-2 (A) and anti-4-1BB Abs (B) for 3 days. Red-dotted boxes denote the dose of rIL-2 and anti-4-1BB Abs that yields the half maximal IFN-γ production. (C) The immune cells were stimulated *in vitro* with either 1 µg/ml E7 CTL epitopes or control Trp2 peptides in the presence of rIL-2 (0.5 ng/ml) and anti-4-1BB Abs (10 ng/ml) for 3 days. (D) Similar experiments in Fig. 7C, except that cRPMI containing rIL-2 and anti-4-1BB Abs was reacted for 1 h 30 min with anti-IL-2 antibodies (50 ng/ml) and 4-1BB-Fc (1 µg/ml) prior to immune cell stimulation. The cell supernatants were used to detect IFN-γ. The values and bars represent the mean INF-γ and SD, respectively. This study was repeated twice with similar results. *p<0.05 using one-way ANOVA compared to non-treatment. **p<0.05 using one-way ANOVA compared to rIL-2 and anti-4-1BB.

We then tested whether combined stimulation through IL-2 and 4-1BB receptors might be directly associated with the increased production of IFN-γ from Ag-specific CD8+ T cells *in vitro*. First, we evaluated recombinant IL-2 (rIL-2) to determine whether it might increase Ag-specific IFN-γ production from the immune cells of mice immunized with E7 DNA vaccines. As shown in Fig. 7A, the addition of rIL-2 to immune cells resulted in increased IFN-γ production in a dose-dependent manner. This increase was detectable when the immune cells were stimulated *in vitro* with E7 peptides but not with Trp2 control peptides. Notably, we previously reported that CD8+ T cells are responsible for E7 CTL peptide-mediated IFN-γ production in the E7 DNA vaccine model [5]. The dose of rIL-2 that yielded the half maximal IFN-γ production was found to be approximately 0.5 ng/ml. Similarly, the addition of anti-4-1BB Abs to the immune cells also resulted in increased IFN-γ production in a dose-dependent fashion (Fig. 7B). In this case, Ag-dependent production of IFN-γ was observed when the immune cells were treated with up to 100 ng/ml of anti-4-1BB Ab. The dose of anti-4-1BB Abs that yielded the half maximal IFN-γ production was found to be approximately 10 ng/ml. When the immune cells were further stimulated *in vitro* with both rIL-2 (0.5 ng/ml) and anti-4-1BB Abs (10 ng/ml), significantly more IFN-γ production was induced from CD8+ T cells in an Ag-dependent manner compared to either treatment alone (Fig. 7C). We further tested whether the increased production of IFN-γ might be mediated by IL-2 and anti-4-1BB Abs by stimulating immune cells *in vitro* with rIL-2 (0.5 ng/ml) and anti-4-1BB Abs (10 ng/ml) in the presence of 50 ng/ml of anti-IL-2 Abs and 1 µg/ml of 4-1BB-Fc (100-fold higher doses of the added amounts of rIL-2 and anti-4-1BB Abs, respectively), and measured IFN-γ levels in the isolated cell supernatants. As shown in Fig. 7D, the addition of anti-IL-2 Abs and 4-1BB-Fc proteins completely blocked IFN-γ production following treatment with rIL-2 and anti-4-1BB, suggesting that this increased production of IFN-γ is indeed mediated by rIL-2 and anti-4-1BB Abs. These data further support the notion that combined stimulation through IL-2 and 4-1BB receptors might be responsible for augmented Ag-specific CTL lytic activity, thus providing better tumor control in this model.

### Augmentation of Ag-specific CTL Lytic Activity *in vivo* by Co-injection of E7 DNA Vaccines with IL-2 cDNA and Anti-4-1BB Abs

**Figure 8:**
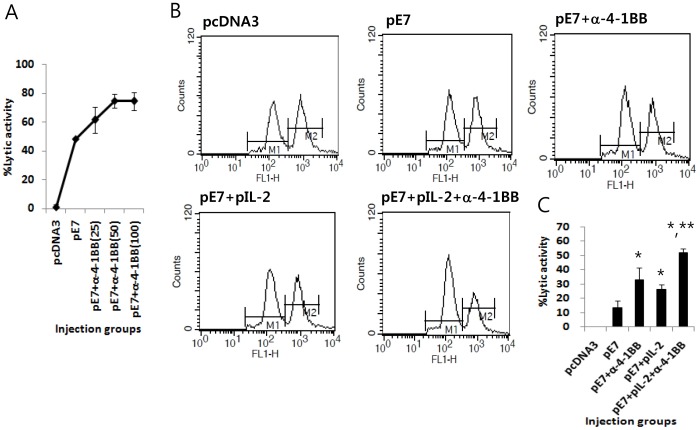
Increased Ag-specific CTL lytic activity *in vivo* by co-injection of E7 DNA vaccines with IL-2 cDNA and anti-4-1BB Abs. (A) Each group of mice (n = 5) was immunized by IM-EP with 50 µg of E7 DNA vaccines (pE7) at 0 and 1 weeks. The animals were also injected i.p. with different doses of anti-4-1BB Abs (25, 50 and 100 µg per mouse) at 0 and 1 weeks. The animals were tested for *in vivo* CTL lytic activity at 2 weeks, as described in “Materials and Methods.” The mice were sacrificed after 10 h and the splenocytes were analyzed by FACS to measure the level of CFSE-labeled cells in each subset. (A) shows the mean % CTL lytic activity of each test group and SD. (B,C) Each group of mice (n = 5) was immunized by IM-EP with either pE7 (50 µg/mouse) or pE7 (50 µg/mouse)+IL-2 cDNA (10 µg/mouse) at 0 and 1 weeks. The animals were also injected i.p. with anti-4-1BB Abs (25 µg/mouse) at 0 and 1 weeks. The animals were tested for *in vivo* CTL lytic activity at 2 weeks, as described in “Materials and Methods.” The mice were sacrificed after 8 h and the splenocytes were analyzed by FACS to measure the level of CFSE-labeled cells in each subset. M1, un-pulsed CFSE low population; M2, E7-pulsed CFSE high population. (C) shows the mean % CTL lytic activity of each test group and SD. *p<0.05 using one-way ANOVA compared to pE7. **p<0.05 using one-way ANOVA compared to pE7+anti-4-1BB or pE7+pIL-2.

We next tested whether co-delivery of E7 DNA vaccines with IL-2 cDNA and anti-4-1BB Abs acts in concert to enhance Ag-specific CTL lytic activity *in vivo.* First, we measured the *in vivo* CTL lytic activity of E7 DNA vaccines (pE7) in relation to different doses of anti-4-1BB Abs. As shown in Fig. 8A, animals injected with pE7 plus different doses of anti-4-1BB Abs showed 49% (0 µg of anti-4-1BB Abs/mouse), 62% (25 µg of anti-4-1BB Abs/mouse), 75% (50 µg of anti-4-1BB Abs/mouse) and 75% (100 µg of anti-4-1BB Abs/mouse) CTL lytic activity. Based on these data, we chose 25 µg of anti-4-1BB Abs per mouse for combined treatment with pE7 because this dose was found to induce approximately 50% maximal CTL activity. Specifically, both 50 and 100 µg of anti-4-1BB Abs appeared to be a saturating dose for the induction of E7-specific CTL lytic activity. Fig. 8B shows CTL lytic activity of animals injected with pE7 in combination with IL-2 cDNA (pIL-2) and anti-4-1BB Abs (25 µg per mouse). As shown in Fig. 8C, animals injected with pE7 had 14% CTL lytic activity. However, animals injected with pE7+anti-4-1BB Abs and pE7+pIL-2 displayed 33% and 26% CTL lytic activity, respectively. Animals injected with pE7+pIL-2+anti-4-1BB Abs had 52% CTL lytic activity. Taken together, these results show that IL-2 cDNA and anti-4-1BB Abs might work together to increase CTL lytic activity induced by E7 DNA vaccines. The *in vivo* CTL data are consistent with the *in vitro* IFN-γ results observed in Fig. 7. Furthermore, these data support the hypothesis that combined stimulation through IL-2 and 4-1BB receptors might be associated with the increased Ag-specific CTL activity induced by E7 DNA vaccines that is responsible for therapeutic antitumor responses.

## Discussion

In the current study, we observed that IL-2 and, to a lesser degree, IL-15 cDNA were effective at increasing the therapeutic antitumor activity induced by E7 DNA vaccines against established TC-1 tumors, as well as enhancing Ag-specific cellular responses. Specifically, tumor (5 mm)-bearing animals treated with E7 DNA vaccines plus IL-2 cDNA displayed a 50% tumor cure rate, whereas animals treated with E7 DNA vaccines alone or E7 DNA vaccines plus IL-15 cDNA showed 0% and 33% tumor cure rates, respectively. This antitumor activity was concomitant with enhanced induction of Ag-specific CTL lytic activity *in vivo* and IFN-γ responses *in vitro*. The animal and *in vitro* data suggest that IL-2 may work slightly better than IL-15 as an E7 DNA vaccine adjuvant, and our data are in agreement with previous reports. For example, IL-2 is known to increase the antitumor efficacy of tumor lysate-primed dendritic cells transferred to mice [33]. Similarly, IL-15 improves the antitumor activity of adoptively transferred CD8+ T cells [34]. IL-15 is stimulatory for the persistence of memory T cells [35], [36]. In this context, IL-15 has been suggested to be a substitute for IL-2 as a vaccine adjuvant in the prevention of cancer and infectious diseases [37]. However, in our large established tumor model where animals die at an early time point, we were unable to evaluate the long-term antitumor memory effects of IL-15 vs. IL-2. Contary to IL-2, IL-15 was not detected in sera even after injecting 50 ug of IL-15 plasmid DNAs. In our subsequent DNA transfection study, IL-2 was detected in both cell lysates and cell supernatnats while IL-15 was detected in cell lysates but not in cell supernatnats, suggesting that IL-15 might be unstable and/or cell surface-attached, making it nearly impossible to detect IL-15 by ELISA. This is supported by previous reports that current methods for detecting IL-15 are limited by the short *in vivo* half-life of IL-15 and the unique way of IL-15 transpresentation [38]–[40]. Our observations showing the superiority of IL-2 cDNA to IL-15 cDNA in induction of Ag-specific cellular and therapeutic antitumor responses correlate well with our previous reports that compared to IL-15 cDNA, IL-2 is better at inducing Ag-specific cellular responses as well as lessening HSV-2-caused diseases when co-delivered as a gD vaccine adjuvant [14]. It is also known that IL-2 and IL-15 provide distinct and contrasting contributions to T cell-mediated immune responses [35], [36]. Furthermore, the tumor-bearing animals in our study treated with E7 DNA vaccines in combination with both IL-2 and IL-15 cDNAs displayed significantly lower therapeutic antitumor activity compared to animals treated with E7 DNA vaccines alone (data not shown), suggesting potential opposing roles of IL-2 and IL-15 in activating T cell responses. However, this needs to be further examined.

We also observed that concurrent therapy using IM-EP of E7 DNA vaccines plus IL-2 cDNA and adoptive transfer of anti-4-1BB Abs augmented antitumor activity against large established TC-1 tumors. For example, tumor-bearing animals treated with the E7 DNA vaccine regimen (E7 DNA vaccines+IL-2 cDNA) displayed a 27% tumor cure rate compared to animals treated with E7 DNA vaccines alone (7%). However, the addition of anti-4-1BB Abs to the E7 DNA vaccine regimen (E7 DNA vaccines+IL-2 cDNA) increased the tumor cure rate from 27% to 67%, a 9.6-fold increase in tumor cure rates following the addition of both IL-2 cDNA and anti-4-1BB Abs. This increased therapeutic antitumor activity positively correlates with Ag-specific CTL lytic activity measured *in vivo*. These data are also concordant with previous reports that Ag-specific CD8+ CTL responses are critical for controlling tumors in TC-1 models [5], [26], [41]–[43]. It is likely that the direct augmentation of Ag-specific CD8+ CTL responses is critical for tumor eradication. In this context, the observed CTL lytic activity appears to be achieved by both IL-2 from IL-2 cDNA through IL-2 receptor signaling combined with anti-4-1BB Ab signaling through 4-1BB receptors. This hypothesis is supported by our *in vitro* results showing that IFN-γ production from E7-specific CD8+ T cells was increased in a synergistic manner by addition of IL-2 and anti-4-1BB Abs and the abolishment of this positive effect when IL-2 and anti-4-1BB Abs were blocked by anti-IL-2 Abs and 4-1BB-Fc proteins, respectively. These data suggest that this response is indeed IL-2- and anti-4-1BB Abs-specific. Consistent with this concept, *in vivo* Ag-specific CTL lytic activity was dramatically increased by co-injection of E7 DNA vaccines with IL-2 cDNA and anti-4-1BB Abs compared to co-injection with either one alone. These *in vitro* and *in vivo* findings are in line with previous findings that agonistic anti-4-1BB Abs activate the CD8+ T cell processes of proliferation, cytokine production and survival [5], [30]–[32]. IL-2 also activates T cell proliferation, cytokine production and survival [12]. However, IL-2 has been known to induce regulatory T cells with immune suppressive functions [44]. Despite this, the IL-2 cDNA used in this study displayed a positive effect on Ag-specific CTL lytic and therapeutic antitumor activity. Moreover, it is possible that the augmentation of antitumor therapeutic activity by IL-2 cDNA and, in particular, anti-4-1BB Abs might be partially associated with the suppression of regulatory T cells by anti-4-1BB Abs. This idea is supported by the recent finding [45]. Here, it is also noteworthy that co-injection of anti-4-1BB Abs contributed to a dramatic decrease in Ag-specific IgG and IgG2b isotype production, supporting the idea that antibodies are not associated with tumor control in this model. In terms of memory responses, concurrent treatment with E7 DNA vaccines+IL-2 cDNA+anti-4-1BB Abs showed a higher induction of memory CD8+ T cell phenotypes (CD44^high^CD8+ T cells) and protective antitumor responses to parental tumor cell re-challenge, as well as increased IFN-γ memory responses to a tumor antigen. The level of this long-term memory response correlates well with the magnitude of Ag-specific CTL lytic and therapeutic antitumor activity achieved by combined treatment with IL-2 cDNA and anti-4-1BB Abs. Aside from CTL augmentation, alteration of the tumor microenvironment also appears to be significant in achieving better tumor control. This is based upon our previous findings that combining therapy using E7 subunit vaccines with chemotherapeutic drugs or radiation made tumor cells more susceptible to CTL-mediated killing, thereby increasing tumor control rates [46], [47]. More recently, it was reported that inhibition of Nanog expression in tumor cells was responsible for increased tumor control by adoptively transferred CTLs [48], further underscoring the importance of modulating the tumor microenvironment for better tumor killing by CTLs. Taken together, our previous and present studies, along with other reports, suggest that augmentation of tumor antigen-specific killer T cell activity and the simultaneous alteration of the tumor microenvironment are likely critical factors in determining therapeutic antitumor efficacy.

It has been reported that the use of IL-2 as an agent for mono-therapy or combination therapy with vaccines can lead to toxic adverse effects, such as capillary leakage [49] and autoimmune toxicity [50]. However, the benefits of IL-2 and IL-15 as therapeutic agents are considered to be dependent solely on their dose, injection schedules and injection timing. In the present study, 10 µg of IL-2 or IL-15 plasmid DNAs delivered by IM-EP had no apparent toxicity or lethality. However, 20 µg of IL-2 or IL-15 plasmid DNAs caused adverse effects in animals after treatment. It is generally accepted that IM-EP can render the myocytes more permeable to DNA, resulting in increased protein production. Thus, it is likely that the levels or duration of expression of IL-2 and IL-15 at the dose of 10 µg by IM-EP may be durable, making it safer to use therapeutically in combination with E7 DNA vaccines.

In conclusion, our data showed that combined stimulation through IL-2 and 4-1BB receptors with IL-2 from IL-2 cDNA and anti-4-1BB Abs, respectively, is critical for increasing tumor cure rates induced by E7 DNA vaccines. This effect appeared to be mediated by the increased induction of Ag-specific CTL lytic activity by direct stimulation of IL-2 and 4-1BB receptors. These results suggest that treatment of tumor-bearing mice with DNA vaccines, in combination with a strong adjuvant and 4-1BB stimulation, is capable of eliciting potent antitumor CTL responses that lead to tumor eradication. These findings could have clinical implications with regards to the design of DNA-based therapeutic vaccines for patients with cancer.
